# Use of Earth’s Magnetic Field for Mitigating Gyroscope Errors Regardless of Magnetic Perturbation

**DOI:** 10.3390/s111211390

**Published:** 2011-11-30

**Authors:** Muhammad Haris Afzal, Valérie Renaudin, Gérard Lachapelle

**Affiliations:** Department of Geomatics Engineering, Schulich School of Engineering, University of Calgary, 2500 University Drive NW, Calgary, AB T2N 1N4, Canada; E-Mails: haris.afzal@ucalgary.ca (M.H.A.); gerard.lachapelle@ucalgary.ca (G.L.)

**Keywords:** pedestrian navigation, orientation estimation, quasi-static magnetic field

## Abstract

Most portable systems like smart-phones are equipped with low cost consumer grade sensors, making them useful as Pedestrian Navigation Systems (PNS). Measurements of these sensors are severely contaminated by errors caused due to instrumentation and environmental issues rendering the unaided navigation solution with these sensors of limited use. The overall navigation error budget associated with pedestrian navigation can be categorized into position/displacement errors and attitude/orientation errors. Most of the research is conducted for tackling and reducing the displacement errors, which either utilize Pedestrian Dead Reckoning (PDR) or special constraints like Zero velocity UPdaTes (ZUPT) and Zero Angular Rate Updates (ZARU). This article targets the orientation/attitude errors encountered in pedestrian navigation and develops a novel sensor fusion technique to utilize the Earth’s magnetic field, even perturbed, for attitude and rate gyroscope error estimation in pedestrian navigation environments where it is assumed that Global Navigation Satellite System (GNSS) navigation is denied. As the Earth’s magnetic field undergoes severe degradations in pedestrian navigation environments, a novel Quasi-Static magnetic Field (QSF) based attitude and angular rate error estimation technique is developed to effectively use magnetic measurements in highly perturbed environments. The QSF scheme is then used for generating the desired measurements for the proposed Extended Kalman Filter (EKF) based attitude estimator. Results indicate that the QSF measurements are capable of effectively estimating attitude and gyroscope errors, reducing the overall navigation error budget by over 80% in urban canyon environment.

## 1. Introduction

The provision to pedestrians of location and orientation information, which in some way can be used for simplifying the task of reaching a particular destination, is called pedestrian navigation. The versatility of environments in which the pedestrian navigation system has to work, differentiates it from land vehicle, sea vessel and aircraft navigation. Of all the possible environments in which the PNS has to work, the urban and indoor environments are the most challenging ones. These challenges arise from the amount and reliability of information available for estimating the navigation parameters. This diversity of environment for pedestrian navigation imposes the use of self-contained sensors for navigation that can provide users with navigation parameters irrespective of the availability of external aids.

### 1.1. Pedestrian Navigation Techniques

Pedestrian navigation can be either accomplished using some man-made information source or by measuring the planetary/universal forces. Radio Frequency (RF) signals are the most widely used man-made information sources for pedestrian navigation [1–4]. The main problem of RF information sources with respect to pedestrian navigation is their reliable availability in all environments. This leads one to the use of planetary/universal information sources for estimating the required parameters for pedestrian navigation. Systems incorporating sensors that can measure planetary/universal forces that can be used for navigation purposes are known as self-contained navigation systems. A well known navigation methodology, namely an Inertial Navigation System (INS), is normally integrated with some extra sensors for such systems. The sensors used for INS mechanization are gyroscopes and accelerometers. INS can be very accurate and reliable depending on the quality of sensors used, but, in the context of pedestrian navigation where cost, size and power consumption dictate sensor selection, these systems are of the lowest accuracy and reliability [5]. In order to improve the navigation solution of a self-contained system, other aiding sensors/information sources are utilized, which can be categorized into the use of additional physical measurements or the specificities of the human walk, *i.e.*, its biomechanics.

The use of sensors additional to gyroscopes and accelerometers constitutes the first category. For example, with magnetometers, the measure of the Earth’s magnetic field can assist the estimation of the direction of motion, which can further be used for estimating errors associated with gyroscopes [6]. But as one moves into urban and indoor environments, the Earth's magnetic field gets perturbed from the man-made infrastructure, rendering it rather useless for absolute orientation estimates.

The second category comprises the use of special constraints dictated by the biomechanical description of the user’s dynamics [7,8]. These include locomotion models, ZUPT and ZARU which can take place whenever the user is stationary or detection of certain events that can be used in conjunction with some gait modeling parameters for estimating the displacements per step. The latter is also known as PDR and is the primary research focus of the pedestrian navigation research community [9]. The main limitations of these techniques are the placement of sensors on the pedestrian’s body. Researchers have concluded that more than 87% of times, the user normally holds the smart-phone in hand while using it for navigation applications [10]. As it is highly unlikely to have zero acceleration or angular rate periods with the sensor block held in hand, this results in a very few ZUPT of ZARU periods, thus rendering the use of special constraints for sensor error estimation rather useless for smart-phone based pedestrian navigation. This leaves one with the PDR approach, which although it can provide promising results for the propagation of location information in various environments, it is unable to solve for the orientation problem, which causes an error growth of third order in position estimates [11].

As is evident from the above discussion, a number of approaches can be taken to target pedestrian navigation. Only one approach seems feasible for seamless navigation in all the pedestrian navigation environments, namely the PDR approach, which utilizes the self-contained navigation systems. Assuming successful detection of gait events, the main limitation of PDR is the orientation estimation. A novel idea has emerged for mitigating the gyroscope errors and estimating the orientation parameters using the Earth’s magnetic field even when it is perturbed by man-made infrastructure [12]. Consequently this new algorithm provides reliable and accurate orientation estimates in diverse and challenging pedestrian navigation environments.

Section 2 introduces the Earth’s magnetic field, its usefulness for orientation estimation, and the effects of indoor environments on it. Section 3 describes different approaches feasible for pedestrians’ attitude estimation, whereas, Section 4 details an Extended Kalman Filter (EKF) based estimator required for mitigation of attitude and sensor errors. The novel technique utilizing perturbed magnetic field for estimating attitude and sensor errors is introduced in Section 5 and Section 6 details the measurement error models required for the EKF. Section 7 addresses the statistical analysis of the proposed mitigation technique. Finally, Section 8 is dedicated to the experimental assessment of the proposed algorithm in a real world environment.

## 2. The Earth’s Magnetic Field

The Earth’s magnetic field is a naturally occurring planetary phenomenon, which can be modeled as a dipole and follows the basic laws of magnetic fields summarized and corrected by Maxwell [13,14]. It is a three dimensional vector originating at the positive pole of the dipole, the magnetic South and ends at the magnetic North pole. For centuries, the Earth’s magnetic field has been successfully used for navigation purposes.

**Figure 1.**
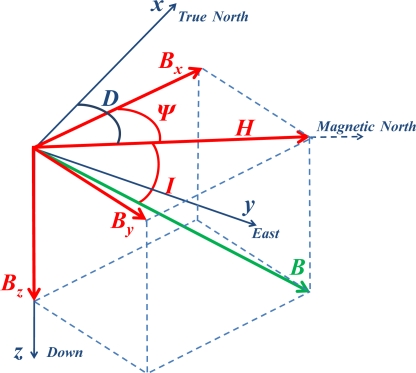
Earth’s magnetic field in Cartesian coordinate system.

With the advancements in sensor technology, this field can now be precisely measured with the help of a sensor commonly known as the magnetometer. With the proper transformation of the field components to the horizontal plane and knowing the declination angle specific to the measurement area and time, a simple trigonometric operation estimates the geographic heading. The magnetic field vector is elaborated in Figure 1. Here ***H*** is the horizontal field component. The angle between the True North and ***H*** is called the declination angle *D* whereas the angle between the magnetic field ***B*** and horizontal plane is called the inclination angle *I. B_x_*, *B_v_* and *B_z_* are the three orthogonal magnetic field components.

### 2.1. Heading Estimation Using the Earth’s Magnetic Field

The orthogonal components of the horizontal magnetic field are used for estimating the heading. Thus the field components must first be transformed to a local level in order to find the horizontal field component of the measured Earth’s magnetic field. For estimating the heading with respect to true North instead of the magnetic North, the declination angle also needs to be predicted using one of the Earth’s magnetic field models [15]. After resolving the magnetic field to the local level and estimating the declination angle, a simple trigonometric relationship is used for estimating the heading from the measured Earth’s magnetic field:

(1) $$\psi ={\text{tan}}^{-1}\left(\frac{B_{y}}{B_{x}}\right)\pm D,$$

### 2.2. Effects of Indoor Environment on Earth’s Magnetic Field

**Figure 2.**
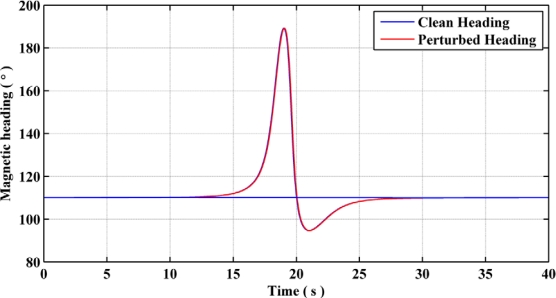
Heading estimates from clean and perturbed magnetic field.

Modeling of the Earth’s magnetic field is possible in an indoor environment in the presence of magnetic dipoles known as magnetic perturbations [12]. These perturbations are due either to electromagnetic devices or magnetization of manmade structures in the presence of an external magnetic field, which is mostly constituted of the Earth’s magnetic field. Figure 2 depicts the heading estimates for a clean and a perturbed environment, while the user walks along a straight line path. A perturbation source is modeled at around the 20 s time mark. Here it can be observed that in the close vicinity of a perturbation source, the heading estimate deviates from the nominal heading with respect to the Earth’s magnetic field, which results from the change in the local magnetic field components due to the perturbation source. If one uses the magnetic heading estimates obtained using the perturbed magnetic field, the effects on pedestrian navigation solution would be adverse.

For better understanding the nature of artificial magnetic perturbations and their influence on Earth’s magnetic field measurements, an exhaustive assessment of the magnetic field was conducted by surveying different indoor and outdoor environments [16]. From these surveys, it has been observed that the effects of perturbation sources on the magnetic heading estimates can cause errors reaching up to 130° in some cases. Thus the urban and indoor magnetic field information cannot be used for effectively estimating ones absolute orientation and new approaches need to be investigated in order to utilize the perturbed magnetic field for pedestrian navigation applications.

## 3. Attitude Computer for Pedestrian Navigation

The digital implementation of the mathematical equations governing the attitude of a body with respect to a reference frame is known as an attitude computer [17]. There are two ways to compute the attitude in three dimensions:

1. using two or more vector measurements,
2. propagating the attitude using angular rate measurements.

### 3.1. Vector Measurements

Using vector measurements for attitude estimation requires finding a common transformation matrix that maps all of the vector measurements in body frame to those in a navigation frame by minimizing the cost function known as Wahba’s problem [18], which is given by:

(2) $$J=\frac{1}{2}\sum_{i=1}^{N}{\left(u_{i}^{b}-C_{n}^{b}u_{i}^{n}\right)}^{T}\;\;\;\left(u_{i}^{b}-C_{n}^{b}u_{i}^{n}\right)$$

where $u_{i}^{b}$ is the *i^th^* vector measurement in the body frame, $u_{i}^{n}$ is the *i^th^* reference vector in the navigation frame and $C_{n}^{b}$ is the transformation matrix that minimizes the cost function *J*. Once this transformation matrix is obtained, the Euler angles can be extracted from it. Vector measurements of at least two non collinear vectors are needed for solving Wahba’s problem [19]. From a pedestrian navigation perspective, mainly two vector measurements are available for this, namely the Earth’s magnetic field vector and the Earth’s gravity vector. But the navigation environments as well as the user dynamics limit the use of these vectors for estimating the attitude. As mentioned earlier, indoor environments are contaminated with magnetic field perturbations. Also, due to the walking dynamics of a pedestrian, with the sensor block in hand, the specific forces measured by the accelerometers will not correspond to those of the gravity vector resolved in the body frame. Therefore the approach of using only non collinear vector measurements for attitude estimation is not feasible for pedestrian navigation.

### 3.2. Angular Rate Measurements

As the vector measurements are not available for estimating the attitude at every epoch for pedestrian navigation, propagation of the attitude in time is necessary. For this purpose, an inertial sensor providing angular rate measurements, namely the rate gyroscope, can be used [20]. Considering the advantages of representing the rotations using a quaternion [12], the quaternion derivative is used for estimating the attitude using angular rate measurements herein, which is given by:

(3) $$\dot{q}=\frac{1}{2}\left[\begin{array}{ccc} -q_{2} & -q_{3} & -q_{4}\\ q_{1} & q_{4} & -q_{3}\\ -q_{4} & q_{1} & q_{2}\\ q_{3} & -q_{2} & q_{1} \end{array}\right]\;\left[\begin{array}{c} -\omega_{x}^{b}\\ -\omega_{y}^{b}\\ -\omega_{z}^{b} \end{array}\right]$$

where $\omega_{x}^{b}$, $\omega_{y}^{b}$ and $\omega_{z}^{b}$ are the three angular rate measurements in the body frame obtained using the rate gyroscopes. *q*_1_, *q*_2_, *q*_3_ and *q*_4_ are the four elements describing the quaternion *q*. In Equation (3), the negative angular rate vector emphasizes the assumption that the transport rate (rate of change of navigation frame) is negligible [20]. This assumption is valid for pedestrian navigation applications involving traveled distances of a few kilometers. As the angular rates required for computing the quaternion derivative are provided by the MEMS rate gyroscopes, the errors associated with them introduce errors in the estimated attitude. The gyroscope errors as well as the attitude errors need to be estimated and mitigated for estimating reliable attitude angles in pedestrian navigation environments using angular rate measurements.

## 4. Extended Kalman Filter for Attitude and Angular Rate Error Estimation

Attitude and its error estimation constitute a non linear problem [21], which can be solved by using a number of estimation approaches. In this article, an extension of a Kalman filter based estimator, namely an Extended Kalman Filter (EKF) is used [22]. The main purpose of this estimator is to model the effects of the gyroscope errors on attitude estimates and use the magnetic field information as corrective measurements to estimate the attitude errors in general and gyroscope errors in particular, which can then be compensated from the present epoch and remodeled for the proceeding ones until new measurements are available. Detailed derivation of the said estimator can be found in a number of books [6,20–25]. Only the appropriate states, measurements and their respective system and measurement error models are detailed herein.

### 4.1. The State Vector

As only the attitude/orientation estimation for pedestrian navigation is targeted herein, the main states to be estimated are the three attitude angles. The attitude is primarily determined using the angular rates obtained using the rate gyroscopes. The deterministic errors associated with this sensor can be compensated for using the calibration parameters [12], leaving the time varying biases as the residual errors. Thus the state vector becomes:

(4) $$x={\left[\begin{array}{cccccc} \varphi & \theta & \psi & b_{\omega_{x}} & b_{\omega_{y}} & b_{\omega_{z}} \end{array}\right]}^{T}={\left[\begin{array}{cc} \Gamma & b_{\omega } \end{array}\right]}^{T}$$

Here, *ϕ* is the roll angle, *θ* is the pitch angle and *Ψ* is the heading angle. The time varying biases associated with the three gyroscopes are represented by *b*_*ω*_*x*__, *b_ω_y__* and *b_ω_z__*.

### 4.2. System Error Model

The perturbed state vector representing errors in Equation (4) is given by:

(5) $$\delta x={\left[\begin{array}{cc} \varepsilon^{n} & \delta b_{\omega } \end{array}\right]}^{T}$$

where $\varepsilon^{n}={\left[\begin{array}{ccc} \varepsilon_{\varphi } & \varepsilon_{\theta } & \varepsilon_{\psi } \end{array}\right]}^{T}$ is the attitude error vector in the navigation frame, which defines the small angle rotations to align the estimated local level frame to the actual one. δ**b***_ω_* are the errors in the inertial sensor bias estimates. As the attitude errors are periodically updated using the measurement vectors, the components of **ε** give the small angle representation of the attitude errors [20]. A small angle transformation matrix (**I**–**E**) can then be used to compensate for the attitude errors from the predicted rotation matrix given by:

(6) $${\hat{C}}_{b}^{n}=\left(I-E\right)\;C_{b}^{n}$$

where $E=\left[\varepsilon^{n}\times \right]=\left[\begin{array}{ccc} 0 & -\varepsilon_{\psi } & \varepsilon_{\theta }\\ \varepsilon_{\psi } & 0 & -\varepsilon_{\varphi }\\ -\varepsilon_{\theta } & \varepsilon_{\varphi } & 0 \end{array}\right]$ is the skew symmetric matrix for the vector **ε** and the circumflex accent on the rotation matrix means that it has been compensated for the attitude errors.

From Equation (6), it can be shown that:

(7) $$C_{b}^{n}=\left(I+E\right)\;{\hat{C}}_{b}^{n}$$

The derivative of Equation (7) is:

(8) $${\dot{C}}_{b}^{n}=\left(I+E\right)\;{\dot{\hat{C}}}_{b}^{n}+\dot{E}{\hat{C}}_{b}^{n}$$

The differential equation for the rotation matrix is given by:

(9) $${\dot{C}}_{b}^{n}=C_{b}^{n}\Omega_{\mathrm{ib}}^{b}$$

where $\Omega_{\mathrm{ib}}^{b}=\left[\omega_{\mathrm{ib}}^{b}\times \right]$ is the skew symmetric matrix of the angular rate vector $\omega_{\mathrm{ib}}^{b}$ obtained from the rate gyroscopes. Substituting Equation (9) in Equation (8) and simplifying gives:

(10) $$\left(I+E\right)\;{\hat{C}}_{b}^{n}\Omega_{\mathrm{ib}}^{b}=\left(I+E\right){\dot{\hat{C}}}_{b}^{n}+\dot{E}{\hat{C}}_{b}^{n}$$

The matrix $\Omega_{\mathrm{ib}}^{b}$ is defined by the uncompensated gyroscope measurements. Let $\delta \Omega_{\mathrm{ib}}^{b}=\left[\delta \omega_{\mathrm{ib}}^{b}\times \right]$ be the skew symmetric matrix for the gyroscope measurement errors. By definition of the different matrices, $\Omega_{\mathrm{ib}}^{b}$ can now be written as:

(11) $$\Omega_{\mathrm{ib}}^{b}={\hat{\Omega }}_{\mathrm{ib}}^{b}+\delta \Omega_{\mathrm{ib}}^{b}$$

Substituting Equation (11) in Equation (10), simplifying and neglecting the second order terms to get a relationship for **Ė** yields:

(12) $$\dot{E}={\hat{C}}_{b}^{n}\delta \Omega_{\mathrm{ib}}^{b}{\hat{C}}_{b}^{s}$$

which, in vector form, becomes:

(13) $${\dot{\varepsilon }}^{n}={\hat{C}}_{b}^{n}\delta \omega_{\mathrm{ib}}^{b}$$

In Equation (13):

(14) $$\delta \omega_{\mathrm{ib}}^{b}=\omega_{\mathrm{ib}}^{b}-{\hat{\omega }}_{\mathrm{ib}}^{b}$$

where $\omega_{\mathrm{ib}}^{b}$ is the true angular rate vector and ${\hat{\omega }}_{\mathrm{ib}}^{b}$ is the angular rate estimates obtained after compensating for the sensor errors, which are estimated using calibration as well as stochastic modeling. The estimated angular rate vector can be written as:

(15) $${\hat{\omega }}_{\mathrm{ib}}^{b}={\tilde{\omega }}_{\mathrm{ib}}^{b}-{\hat{b}}_{\omega }$$

where ${\tilde{\omega }}_{\mathrm{ib}}^{b}$ is the raw angular rate measurement vector and **b̂***_ω_* is the estimate of the time varying bias vector obtained from the estimator (EKF). Substituting for the gyroscope model, with **ν***_ω_* being the wideband noise, and using Equation (15) in Equation (14), one gets:

(16) $$\delta \omega_{\mathrm{ib}}^{b}={\tilde{\omega }}_{\mathrm{ib}}^{b}-b_{\omega }-\nu_{\omega }-{\tilde{\omega }}_{\mathrm{ib}}^{b}+{\hat{b}}_{\omega }$$

which after simplification becomes:

(17) $${\delta \omega }_{\mathrm{ib}}^{b}=-{\delta b}_{\omega }-\nu_{\omega }$$

where δ**b***_ω_* = **b***_ω_* – **b̂***_ω_* is the perturbation of the time varying gyroscope biases. Equation (13) now becomes:

(18) $${\dot{\varepsilon }}^{n}=-{\hat{C}}_{s}^{n}\delta b_{\omega }-{\hat{C}}_{s}^{n}v_{\omega }$$

The time varying gyroscope bias is modeled as an exponentially correlated noise term, resulting in the derivative of perturbations in the time varying gyroscope bias δ**b***_ω_* given by [20]:

(19) $$\delta {\dot{b}}_{\omega }=-\frac{1}{\underset{F_{\omega }}{\underset{︸}{\beta_{b_{\omega }}}}}\delta b_{\omega }+\nu_{b_{\omega }}$$

where ***β***_**b**_*ω*__ is the correlation time and ***ν***_**b**_*ω*__ is the noise vector for the stochastic modeling of the time varying gyroscopes’ biases. Equations (18) and (19) lead to the following system dynamics model:

(20) $$\underset{\delta x}{\underset{︸}{\left[\begin{array}{c} {\dot{\varepsilon }}^{n}\\ \delta {\dot{b}}_{\omega } \end{array}\right]}}=\underset{F}{\underset{︸}{\left[\begin{array}{cc} 0 & -{\hat{C}}_{b}^{n}\\ 0 & F_{\omega } \end{array}\right]}}\underset{\delta x}{\underset{︸}{\left[\begin{array}{c} \varepsilon^{n}\\ \delta b_{\omega } \end{array}\right]}}+\underset{G}{\underset{︸}{\left[\begin{array}{cc} -{\hat{C}}_{b}^{n} & 0\\ 0 & I \end{array}\right]}}\underset{w}{\underset{︸}{\left[\begin{array}{c} \nu_{\omega }\\ \nu_{b_{\omega }} \end{array}\right]}}$$

where ***F*** is the dynamics matrix, ***G*** is the shaping matrix and ***w*** is the system noise matrix as defined in literature on Kalman filter.

### 4.3. Measurement Error Models

The measurements used for compensating the errors associated with the attitude vector in general and gyroscope biases in particular are herein solely based on the magnetic field vector measurements. In order to utilize the clean as well as perturbed magnetic field measurements, a novel technique for estimating the sensor frame’s angular rates using a tri-axis magnetometer was developed and is detailed in the following section.

## 5. Quasi-Static Field (QSF) Based Attitude and Angular Rate Measurements (Patent Pending)

The novel idea for estimating attitude and gyroscope errors using magnetic field measurements for pedestrian navigation environments mainly involves detecting quasi-static total magnetic field periods during pedestrian motion and utilizing them as measurements for estimating attitude and gyroscope errors. Indeed when the local magnetic field is quasi-static, the rate of change of the magnetic field is combined with the rotational rate of change of the inertial device generating an estimated gyroscope error, which can be further used to correct for time-varying inherent gyroscope errors [12]. Contrary to existing solutions, this technique is working in magnetically perturbed environments as long as the field is identified as constant over a selective period of time. Further, in order to successfully detect the presence of QSF periods, proper pre-calibration of the magnetic field sensors is necessary. This is achieved by utilizing a calibration algorithm developed by the authors [26]. The QSF detector, developed using statistical signal processing techniques, is now presented.

The Earth’s magnetic field, though a good source of information for estimating heading outdoor, suffers severe degradations in the indoors caused by magnetic field perturbations [16]. These perturbations are of changing magnitudes and directions, which induce random variations in the total magnetic field. These variations render the magnetic field information useless for absolute orientation estimation with respect to the magnetic North in indoor environments. Although the magnetic field indoor is not spatially constant due to changing perturbation sources, depending on the pedestrian’s speed and surroundings, it is possible to have locations as well as short periods (user not moving) when the perturbed magnetic field is constant in magnitude as well as in direction. The rate of change of the total magnetic field in such situations will be ideally zero. It is possible to have very slight changes in the magnitude and direction of the total magnetic field (due to sensor noise) that can still be considered as quasi-static. Thus information to be considered for detecting a QSF is the rate of change of the total magnetic field ||**Ḃ**||, which is referred to as the field gradient and is computed using:

(21) $$\left\|\dot{B}\right\|=\left\|\frac{B_{k}-B_{k-1}}{\Delta t}\right\|$$

where **B***_k_* is the magnetic field at the current epoch, **B***_k−1_* is the magnetic field at the previous epoch and Δ*t* is the measurement update rate. For a window of size *N*, a QSF detector will detect a static field if:

(22) $${\left\|{\dot{B}}_{k}\right\|}_{k=n}^{n+N-1}\approx 0$$

Let the hypothesis for a non-static field be *H_0_* and that for a quasi-static field be *H_1_* respectively. The Probability Density Functions (PDFs) associated with these two hypotheses are:

(23) $$\begin{array}{l} f\left(\left\|{\dot{B}}_{k}\right\|;H_{0}\right)\\ f\left(\left\|{\dot{B}}_{k}\right\|;H_{1}\right) \end{array}$$

The rate of change of the total magnetic field is also contaminated by white Gaussian noise ***v***_*k*_, which, when modeled with the measurements, gives:

(24) $$y_{k}=\left\|{\dot{B}}_{k}\right\|+v_{k}$$

where *y_k_* is the information to be tested for *H_0_* or *H_1_*. Under *H_0_*, ||**Ḃ***_k_*|| is the unknown parameter required to describe the signal completely. Therefore, for the two hypotheses, ||**Ḃ***_k_*|| is defined as:

(25) $$\begin{array}{l} H_{0}:\exists k\in \Omega_{n}\;\text{s.t.}\;\;\;\;\;\left\|{\dot{B}}_{k}\right\|\neq 0\\ H_{1}:\forall k\in \Omega_{n}\;\text{then}\;\;\;\left\|{\dot{B}}_{k}\right\|=0 \end{array}$$

where **Ω**_n_ = {l ∈ N: *n* ≤ l ≤ *n* + *N* − 1} with N ∈ N and n ∈ N.

As the complete knowledge about ||**Ḃ***_k_*|| is unknown for *H_0_*, the PDF in this case is given by:

(26) $$f\left(y;\left\|{\dot{B}}_{k}\right\|,\;H_{0}\right)=\prod_{k\in \Omega_{n}}\frac{1}{\sqrt{2\pi \sigma_{\left\|{\dot{B}}_{k}\right\|}^{2}}}\;\exp\;\left(\frac{-1}{2\sigma_{\left\|{\dot{B}}_{k}\right\|}^{2}}{\left(y_{k}-\left\|{\dot{B}}_{k}\right\|\right)}^{2}\right)$$

Let the Maximum Likelihood Estimator (MLE) for the unknown parameter in case of *H_0_* be $\left\|{\hat{\dot{B}}}_{k}\right\|$, which is given by the mean of the signal as:

(27) $$\left\|{\hat{\dot{B}}}_{k}\right\|=\frac{1}{N}\sum_{k\in \Omega_{n}}\;y_{k}$$

Now the PDF for *H*_0_ becomes:

(28) $$f\left(y;\left\|{\hat{\dot{B}}}_{k}\right\|,\;H_{0}\right)=\prod_{k\in \Omega_{n}}\frac{1}{\sqrt{2\pi \sigma_{\left\|{\dot{B}}_{k}\right\|}^{2}}}\;\exp\;\left(\frac{-1}{2\sigma_{\left\|{\dot{B}}_{k}\right\|}^{2}}{\left(y_{k}-\left\|{\hat{\dot{B}}}_{k}\right\|\right)}^{2}\right)$$

For hypothesis *H*_1_, the rate of change of the total magnetic field is known (it will be zero), therefore the PDF in this case becomes:

(29) $$f\left(y;\;H_{1}\right)=\prod_{k\in \Omega_{n}}\frac{1}{\sqrt{2\pi \sigma_{\left\|{\dot{B}}_{k}\right\|}^{2}}}\;\exp\;\left(\frac{-1}{2\sigma_{\left\|{\dot{B}}_{k}\right\|}^{2}}\;y_{k}^{2}\right)$$

The Generalized Likelihood Ratio Test (GLRT) for detecting a quasi-static field is given by:

(30) $$\wedge \left(y\right)=\frac{f\left(y;\left\|{\hat{\dot{B}}}_{k}\right\|,\;H_{0}\right)}{f\left(y;\;H_{1}\right)}<\lambda$$

Substituting for the PDFs in Equation (30) and simplifying, one gets:

(31) $$\wedge \left(y\right)=\prod_{k\in \Omega_{n}}\;\exp\;\left(\frac{1}{\alpha_{\left\|{\dot{B}}_{k}\right\|}^{2}}\;y_{k}\left\|{\hat{\dot{B}}}_{k}\right\|-\frac{1}{2\sigma_{\left\|{\dot{B}}_{k}\right\|}^{2}}{\left\|{\hat{\dot{B}}}_{k}\right\|}^{2}\right)<\lambda$$

Taking the natural log on both sides and simplifying yields:

(32) $$\left|\frac{1}{\sqrt{N}}\left(\sum_{k\in \Omega_{n}}\;y_{k}\right)\right|<\gamma_{\left\|{\dot{B}}_{k}\right\|}$$

where $\gamma_{\left\|{\dot{B}}_{k}\right\|}=\sqrt{2\sigma_{\left\|{\dot{B}}_{k}\right\|}^{2}\ln\left(\lambda \right)}$ is the threshold for QSF detection.

## 6. Use of QSF Detected Periods for Attitude and Gyroscope Error Estimation

Once the QSF periods are detected during pedestrian motion, the next step is to utilize the magnetic field information during such periods for estimating the attitude angles and the gyroscope errors. This section derives the equations required for using QSF magnetic field measurements, even perturbed, in the EKF developed in Section 4. It principally consists of a measurement error model that is used for updating the state vector of the navigation filter.

### 6.1. QSF Measurement Error Model Using the Local Magnetic Field

Let the magnetic field measurement in the sensor frame at the start, *i.e.*, the *k*^th^ epoch, of the quasi-static field period be given by:

(33) $$B_{{\mathrm{QSF}}_{k}}^{b}={\left[\begin{array}{ccc} B_{{\mathrm{QSF}}_{x}}^{b} & B_{{\mathrm{QSF}}_{y}}^{b} & B_{{\mathrm{QSF}}_{z}}^{b} \end{array}\right]}^{T}$$

Considering the attitude at the start of quasi-static period as the reference for the measurement model, the magnetic field measurement can be transformed to the navigation frame using:

(34) $$B_{{\mathrm{QSF}}_{k}}^{n}={\hat{C}}_{b}^{n}B_{{\mathrm{QSF}}_{k}}^{b}$$

$B_{{\mathrm{QSF}}_{k}}^{n}$ is considered as a measurement during quasi-static field periods. Indeed in the novel approach the magnetic field information extracted from a geomagnetic field model is not considered as a measurement of the truth but rather the field $B_{{\mathrm{QSF}}_{k}}^{n}$ is considered as a reference over the QSF period. As the inaccuracies in the sensor will bias the estimated attitude, the transformations of proceeding magnetic field measurements from body to navigation frame using the updated ${\hat{C}}_{b}^{n}$ would be different from $B_{{\mathrm{QSF}}_{k}}^{n}$ hence introducing the measurement error. This gives the relationship for the first measurement error model:

(35) $$\delta B_{{\mathrm{QSF}}_{k}}^{n}=B_{{\mathrm{QSF}}_{k}}^{n}-{\hat{C}}_{b}^{n}B_{{\mathrm{QSF}}_{k}}^{b}$$

At the *k*^th^ epoch, Equation (35) would equal to zero as $B_{{\mathrm{QSF}}_{k}}^{n}$ is obtained using ${\hat{C}}_{b}^{n}B_{{\mathrm{QSF}}_{k}}^{b}$. Thus the magnetic field information from the proceeding epoch along with new attitude estimate for ${\hat{C}}_{b}^{n}$ are needed. Let $B_{{\mathrm{QSF}}_{k+1}}^{b}$ be the next magnetometer measurement while the QSF period lasts. Substituting the new magnetic field measurement, at epoch *k + 1*, in Equation (35), the measurement error model becomes:

(36) $$\delta B_{{\mathrm{QSF}}_{k+1}}^{n}=B_{{\mathrm{QSF}}_{k}}^{n}-{\hat{C}}_{b}^{n}B_{{\mathrm{QSF}}_{k+1}}^{b}$$

Ideally, as the magnetic field during QSF period is locally static (magnetic field vector not changing its magnitude or direction), Equation (36) should be equal to zero. But due to errors in rate gyroscope measurements, which are used for estimating the rotation matrix, the following perturbed model is obtained:

(37) $$\delta B_{{\mathrm{QSF}}_{k+1}}^{n}=B_{{\mathrm{QSF}}_{k}}^{n}-\left(I-E\right)\;C_{b}^{n}\left(B_{{\mathrm{QSF}}_{k+1}}^{b}+\eta_{B}\right)$$

where **η_B_** is the measurement noise of the magnetometers.

Simplifying (37) to get a relationship between measurements and states, one obtains:

(38) $$\delta B_{{\mathrm{QSF}}_{k+1}}^{n}=B_{{\mathrm{QSF}}_{k}}^{n}-B_{{\mathrm{QSF}}_{k+1}}^{n}-C_{b}^{n}\eta_{B}+EB_{{\mathrm{QSF}}_{k+1}}^{n}+EC_{s}^{n}\eta_{B}$$

Because the last term in Equation (38) is of the second order in errors, neglecting it results in:

(39) $$\delta B_{{\mathrm{QSF}}_{k+1}}^{n}=-\left[B_{{\mathrm{QSF}}_{k+1}}^{n}\times \right]\varepsilon^{n}-C_{b}^{n}\eta_{B},$$

where $\left[B_{{\mathrm{QSF}}_{k+1}}^{n}\times \right]-\left[\begin{array}{ccc} 0 & -B_{{\mathrm{QSF}}_{z}}^{n} & B_{{\mathrm{QSF}}_{y}}^{n}\\ B_{{\mathrm{QSF}}_{z}}^{n} & 0 & -B_{{\mathrm{QSF}}_{x}}^{n}\\ -B_{{\mathrm{QSF}}_{y}}^{n} & B_{{\mathrm{QSF}}_{x}}^{n} & 0 \end{array}\right]$ is the skew symmetric matrix of vector $B_{{\mathrm{QSF}}_{k+1}}^{n}$.

From Equation (39), the first QSF measurement error model becomes:

(40) $$\delta B_{{\mathrm{QSF}}_{k+1}}^{n}=\left[\begin{array}{cc} -\left[B_{{\mathrm{QSF}}_{k+1}}^{n}\times \right] & 0_{1\times 3} \end{array}\right]\left[\begin{array}{c} \varepsilon^{n}\\ \delta b_{\omega } \end{array}\right]-\left[\begin{array}{cc} C_{b}^{n} & 0\\ 0 & 0 \end{array}\right]\;\left[\begin{array}{c} \eta_{B}\\ 0 \end{array}\right]$$

### 6.2. QSF Measurement Error Model Using the Rate of Change of the Local Magnetic Field

During the quasi-static field periods, the rate of change of the reference magnetic field is zero. Using this information as a measurement, one gets:

(41) $$\begin{array}{l} B_{{\mathrm{QSF}}_{k}}^{n}={\hat{C}}_{b}^{n}B_{{\mathrm{QSF}}_{k+1}}^{b}\\ {\hat{C}}_{n}^{b}B_{{\mathrm{QSF}}_{k}}^{n}=B_{{\mathrm{QSF}}_{k+1}}^{b} \end{array}$$

Taking the derivative of Equation (41) to get the relationship between the rate of change of a vector in two different frames [28], one gets:

(42) $${\hat{C}}_{b}^{b}{\dot{B}}_{{\mathrm{QSF}}_{k}}^{n}={\dot{B}}_{{\mathrm{QSF}}_{k+1}}^{b}+\omega_{B}^{b}\times B_{{\mathrm{QSF}}_{k+1}}^{b}$$

where $\omega_{B}^{b}={\left[\begin{array}{ccc} \omega_{B_{x}} & \omega_{B_{y}} & \omega_{B_{z}} \end{array}\right]}^{T}$ is the angular rate vector required for rotating the magnetic field measurements between two epochs in the sensor frame. Because the QSF periods are identified as those where the field vector in the navigation frame is not changing its magnitude and orientation, the left hand side of Equation (42) equals to zero, reducing the equation to:

(43) $${\dot{B}}_{{\mathrm{QSF}}_{k+1}}^{b}=-\omega_{B}^{b}\times B_{{\mathrm{QSF}}_{k+1}}^{b}.$$

During user motion, the magnetic field components in the body frame will encounter changes, which can be modeled by Equation (43). But due to errors in gyroscopes angular rates, the predicted changes in magnetic field will be different from the measured ones given by:

(44) $$\delta {\dot{B}}_{{\mathrm{QSF}}_{k+1}}^{b}={\dot{B}}_{{\mathrm{QSF}}_{k+1}}^{b}-{\dot{\hat{B}}}_{{\mathrm{QSF}}_{k+1}}^{b}$$

where B˙QSFk+1b=(BQSFk+1b−BQSFkb)Δt and Δt is the time period between two consecutive epochs. Expanding Equation (44) and substituting from Equation (43) results in:

(45) $$\begin{array}{ll} \delta {\dot{B}}_{{\mathrm{QSF}}_{k+1}}^{b} & ={\dot{B}}_{{\mathrm{QSF}}_{k+1}}^{b}-{\dot{\hat{B}}}_{{\mathrm{QSF}}_{k+1}}^{b}\\ & ={\dot{B}}_{{\mathrm{QSF}}_{k+1}}^{b}+{\hat{\omega }}_{B}^{b}\times \left(B_{{\mathrm{QSF}}_{k+1}}^{b}+\eta_{B}\right)\\ & ={\dot{B}}_{{\mathrm{QSF}}_{k+1}}^{b}+\left(\omega_{B}^{b}+\delta \omega_{B}^{b}\right)\times B_{{\mathrm{QSF}}_{k+1}}^{b}+{\hat{\omega }}_{B}^{b}\times \eta_{B}\\ & ={\dot{B}}_{{\mathrm{QSF}}_{k+1}}^{b}+\omega_{B}^{b}\times B_{{\mathrm{QSF}}_{k+1}}^{b}+\delta \omega_{B}^{b}\times B_{{\mathrm{QSF}}_{k+1}}^{b}+{\hat{\omega }}_{B}^{b}\times \eta_{B} \end{array}$$

The first two terms give the rate of change of the reference magnetic field, which, during QSF periods, is zero. $\delta \omega_{B}^{b}$ is the error in $\omega_{B}^{b}$ caused by the gyroscope biases. Thus Equation (45) reduces to:

(46) $$\delta {\dot{B}}_{{\mathrm{QSF}}_{k+1}}^{b}=\delta \omega_{B}^{b}\times B_{{\mathrm{QSF}}_{k+1}}^{b}+{\hat{\omega }}_{B}^{b}\times \eta_{B}$$

which can be rewritten as:

(47) $$\delta {\dot{B}}_{{\mathrm{QSF}}_{k+1}}^{b}=-\left[B_{{\mathrm{QSF}}_{k+1}}^{b}\times \right]\delta \omega_{B}^{b}+\left[{\hat{\omega }}_{B}^{b}\times \right]\eta_{B}$$

giving the following measurement model:

(48) $$\delta {\dot{B}}_{{\mathrm{QSF}}_{k+1}}^{b}=\left[\begin{array}{cc} 0_{3\times 3} & -\left[{\dot{B}}_{{\mathrm{QSF}}_{k+1}}^{b}\times \right] \end{array}\right]\;\left[\begin{array}{c} \varepsilon^{n}\\ \delta b_{\omega } \end{array}\right]+\left[\begin{array}{cc} 0_{1\times 3} & 0_{1\times 3}\\ 0_{1\times 3} & \left[{\hat{\omega }}_{B}^{b}\times \right] \end{array}\right]\;\left[\begin{array}{c} 0_{3\times 1}\\ \eta_{B} \end{array}\right]$$

### 6.3. Full QSF Measurement Error Model

Combining Equations (40) and (48), the complete measurement error model using QSF is:

(49) $$\underset{\delta z}{\underset{︸}{\left[\begin{array}{c} \delta B_{{\mathrm{QSF}}_{k+1}}^{n}\\ \delta {\dot{B}}_{{\mathrm{QSF}}_{k+1}}^{b} \end{array}\right]}}=\underset{H_{\mathrm{QSF}}}{\underset{︸}{\left[\begin{array}{cc} -\left[B_{{\mathrm{QSF}}_{k}}^{n}\times \right] & 0_{3\times 3}\\ 0_{3\times 3} & -\left[{\dot{B}}_{{\mathrm{QSF}}_{k+1}}^{b}\times \right] \end{array}\right]}}\underset{\delta x}{\underset{︸}{\left[\begin{array}{c} \varepsilon^{n}\\ \delta b_{\omega } \end{array}\right]}}+\left[\begin{array}{c} -C_{b}^{n}\\ {\hat{\omega }}_{B}^{b}\times \end{array}\right]\eta_{B},$$

which can be utilized for constraining the error growth in attitude angles and estimating the rate gyroscope errors.

## 7. Statistical Analysis of the QSF Detector

In order to quantify the performance of the proposed quasi-static magnetic field detector, statistical analysis was conducted. From Equation (32), it can be observed that there are a number of tuning parameters that need to be evaluated for effectively using the QSF detector. These are the threshold, noise variance and the number of samples (window size) required for the detection test statistics.

### 7.1. Measurement Noise Variance

**Figure 3.**
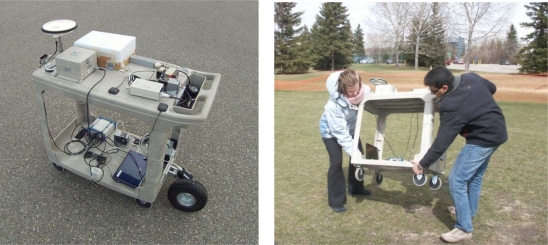
Test setup for the magnetic field survey including the Bartington fluxgate and the SPAN CPT HG1700 INS System from NovAtel.

**Figure 4.**
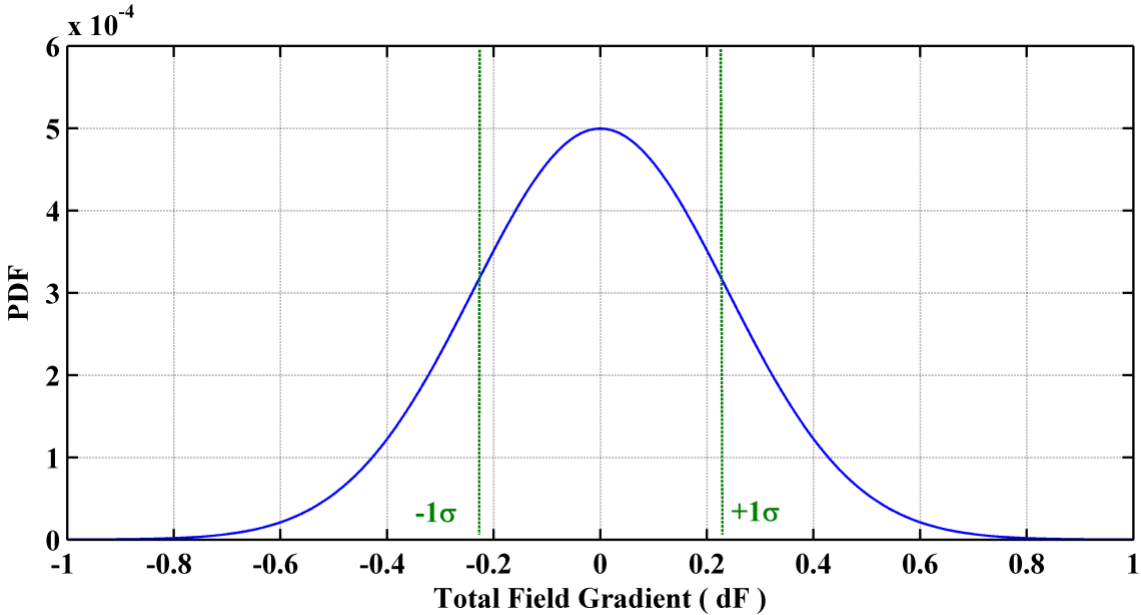
Probability Density Function (PDF) of total field gradient during constant field periods.

This factor corresponds to the variance of the total field gradient when the field itself is not changing. This gives a measure of the gradient noise that is encountered during quasi-static field periods. More than seven hours of data in five different environments that are common for pedestrian navigation have been collected and analyzed for evaluating this parameter [16,27]. The magnetic field survey was conducted using a tri-axis Bartington high resolution and high sensitivity fluxgate magnetometer. The magnetically derived heading was compared with the true heading estimated by post-processing synchronous measurements collected with the tactical grade inertial system (INS): the SPAN-CPT HG1700 from NovAtel. As shown in Figure 3, all devices were rigidly mounted on a plastic cart. The complete hardware setup was calibrated by performing 3D rotational maneuvers in a perturbation free environment and applying the complete calibration algorithm detailed in [26] to the recorded data. Figure 4 depicts the derived field gradient noise distribution, which is used for estimating the noise variance at 1σ. This comes out to be 0.057 μT^2^.

### 7.2. Selection of Threshold and Window Size Using Receiver Operating Characteristics

**Figure 5.**
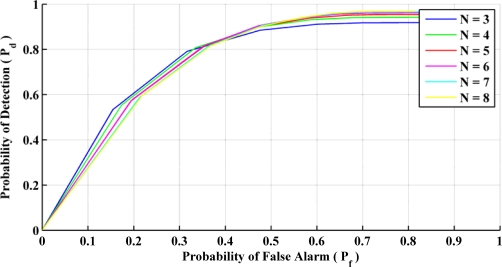
ROC for different window sizes.

The Receiver Operating Characteristics (ROC) curve allows one to select the test statistics acceptance threshold based on the required probability of detection *P_d_* and the acceptable probability of false alarm *P_f_*. Figure 5 shows the ROC for the QSF detector for different sample window sizes. The sensor sampling rate is 0.04 s, which gives a minimum window size of 0.12 s and a maximum of 0.32 s in this case. It can be observed that the ROC tends to flatten out after *P_d_* = 0.8. Thus selecting a *P_d_* any larger than this value will cause more false alarms. Hence a *P_d_* of approximately 0.8 is selected for this detector. The effect of the window size on the detector’s performance is negligible at the selected *P_d_*. Therefore a window size of three samples is selected to reduce the processing burden. Table 1 summarizes the parameters selected for the QSF detector.

### 7.3. Occurrence and Durations of QSF Periods in Magnetically Perturbed Environments

**Figure 6.**
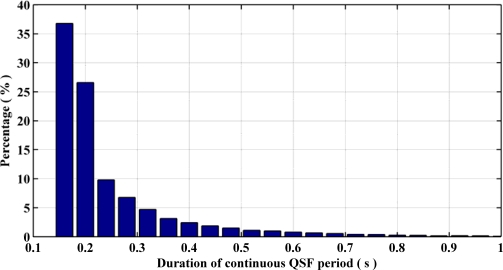
Continuous QSF periods and their occurrence.

**Figure 7.**
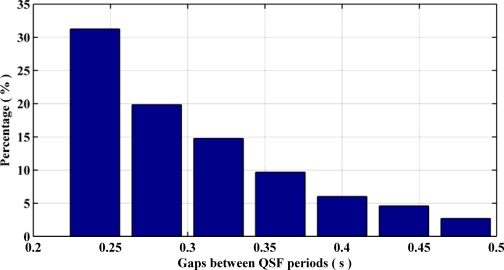
Duration of gaps between QSF periods.

Figure 6 summarizes the QSF detection periods and their respective durations for different pedestrian navigation environments surveyed. Most of the detection periods have a duration of 120 ms to 300 ms. Figure 7 depicts the shortest and longest gaps between two consecutive QSF periods and their percentages of occurrence respectively. The minimum gap, *i.e.*, 240 ms, occurs more frequently as compared with the maximum gap of 480 ms. Therefore it can be concluded that the QSF periods are encountered frequently and hence may allow for estimation of angular rate errors. It is worth mentioning here that unlike some pedestrian navigation applications where Zero Velocity Updates (ZUPT) occur frequently during a pedestrian’s walk (e.g., shoe mounted sensors), when the sensor block is in the hand or in a pocket/purse, these may not be encountered at all. In such scenarios, QSF periods can still be used effectively for providing regular measurements for sensor error estimation.

## 8. Experimental Assessment of the Proposed Attitude Computer

In order to assess the performance of the proposed QSF based orientation/attitude estimation algorithm, test data was collected in a real world environment, which is most common for pedestrian navigation applications: downtown. The test setup used for analyzing the impact of the proposed algorithm on attitude estimation comprised a Multiple Sensor Platform (MSP) and an optical wheel encoder developed by the author [12]. The MSP comprises a tri-axis gyroscope made of a dual-axis ST Microelectronics’ LPR530AL and a single-axis LY530ALH from the same manufacturer. It includes a HMC5843 tri-axis Anisotropic Magneto-Resistive (AMR) sensor for magnetic field measurements. Finally a tri-axis Analog Devices’ ADXL335 accelerometer completes the inertial measurement unit. The wheel encoder is used here for measuring the pedestrian’s walking speed so as to bring the outcome of the proposed algorithm from attitude domain to the position domain and provide better insight into the performance of the system. The wheel encoder is capable of computing the pedestrian’s walking speed with an accuracy of ±4 × 10^−3^ m/s. This walking speed is later resolved into North and East components using the estimated attitude to compute the position, the latter being obtained by integrating the velocity components. As this article focuses only on attitude estimation, the wheel encoder provides accurate speed measurements, which are necessary for de-correlating the velocity error budget from the attitude one, allowing the assessment of attitude accuracies only.

In an actual portable device such as a smart-phone, the walking speed would be measured by accelerometers. Although smart-phones of today are equipped with accelerometers, performing gait analysis with a handheld device is a challenging task and constitutes a research topic in itself. Thus with the use of wheel encoder, the experimental assessments of the proposed attitude estimator in the position domain as described herein, can be considered free of errors induced by speed sensors (accelerometers), providing a better insight into attitude accuracy. However the MSP developed for this research is hosting a tri-axis of accelerometers, which can be used in future for investigating different methods to estimate stride length and speed. This work targets the implementation of a complete pedestrian navigation system.

**Figure 8.**
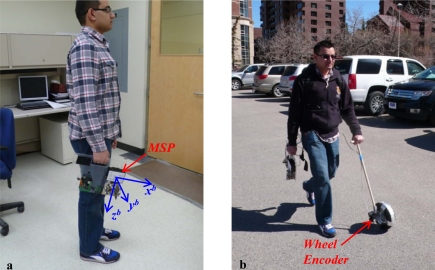
Test data collection setup.

The MSP was rigidly mounted on a plastic plate, which can be easily carried in a hand. The wheel encoder is mounted on a pole that can be held by a pedestrian and pushed along the ground for measuring the walking speed. Figure 8(a) shows the handheld arrangement of the sensor module used for the test data collection. Here the pedestrian and body frames are also identified to clarify in which frame (body frame) the attitude is estimated. Figure 8(b) shows the overall test data collection setup including the wheel encoder. It is assumed that the body frames x–z plane is aligned with that of the pedestrian frame in order to account for the ambiguity between sensor frame’s orientation and users walking direction. This is achieved with the help of the hand held plastic plate. Thus the sensor’s frame is effectively aligned with the body frame.

### 8.1. Assessment Criterion

In order to assess the impact of the proposed algorithm on attitude estimation for pedestrian navigation, the solution repeatability criterion is chosen. Multiple paths of the same trajectory were followed in different environments keeping the same starting and ending point to assess the performance. The paths were followed so as to keep the separation between them within one metre if possible. This is achieved by following prominent patterns on the ground (tiles boundaries, pavement markings/intersections *etc*.).

### 8.2. Test Environment

In order to assess the impact of the proposed algorithms on attitude estimation for pedestrian applications, a urban canyon was selected.

**Figure 9.**
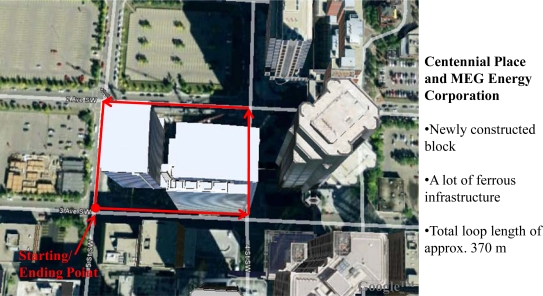
Downtown Calgary data collection environment.

Urban canyons can be considered as one of the regions where pedestrian navigation applications have a lot of commercial significance. Also before moving indoor, one often ends up being in an urban canyon for some time. Hence detailed analysis of the proposed algorithm in this environment is very important. Figure 9 gives the bird’s eye view of the test region selected in downtown Calgary for the assessment. The block selected is newly constructed with a walkway filled with ferrous infrastructure all around including phone booths, newspaper dispensers, street light poles and manholes. The walking trajectory around this block was approximately 370 m and was traversed thrice for repeatability testing.

**Figure 10.**
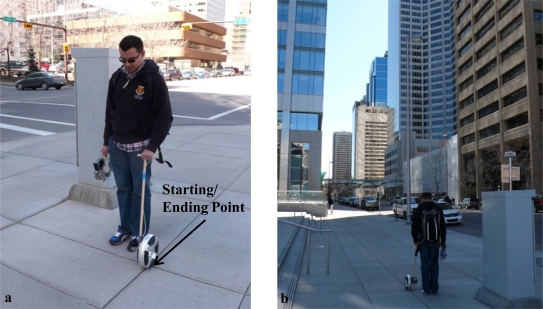
Data collection in downtown Calgary.

Figure 10(a) shows the same starting and ending point for each loop around the block with a traffic signal control panel right beside it. The last was made out of metal and hence contributed to the magnetic field perturbations in this region. Figure 10(b) shows one of the paths traversed in the selected region with high rise buildings and metallic infrastructure all around. Indeed this environment includes numerous magnetic field perturbation sources and hence can be considered a good test area for assessing the attitude estimation algorithms presented in this article.

### 8.3. Urban Canyon Test Results

**Figure 11.**
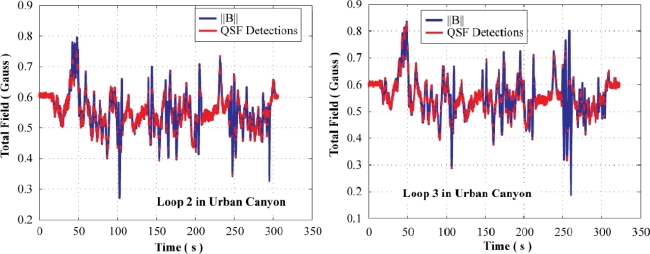
Total field and QSF detections for similar paths in urban canyon.

Figure 11 shows the total field observed in the urban canyon environment along with the QSF detections. It can be observed that the overall signatures of the total field as well as the detection of QSF periods are temporally very similar for different paths. This is because the paths traversed were kept within 1 m of one another for assessing the repeatability of the results.

**Figure 12.**
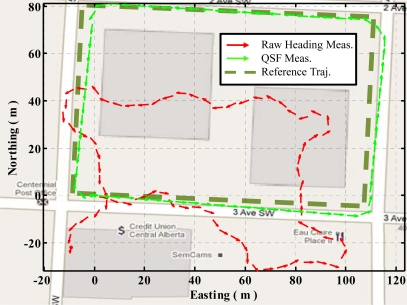
Raw heading measurements versus QSF measurements.

Figure 12 shows the trajectories obtained using raw heading versus QSF measurements for estimating gyroscope errors. It can be observed that due to severe magnetic field perturbations, the trajectory obtained using raw heading estimates has a maximum error of 43 m as compared to 5 m in case of QSF measurements.

**Figure 13.**
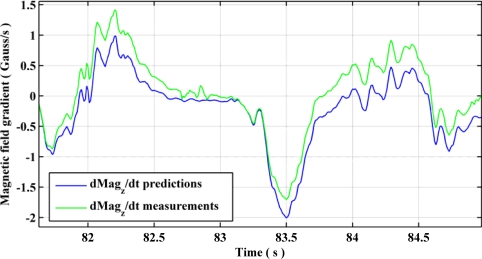
Estimated *versus* actual rate of change of magnetic field component before gyroscope bias estimation.

**Figure 14.**
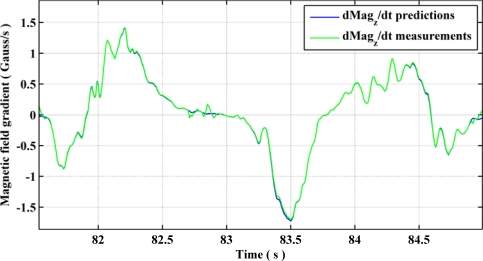
Estimated *versus* actual rate of change of magnetic field component after gyroscope bias estimation.

The observability of gyroscope biases using QSF measurements is presented graphically in Figures 13 and 14. Using Equation (43), one can obtain the estimates of the rate of change of magnetic field components using gyroscopes during a QSF period. Due to presence of biases in the gyroscope measurements, these estimates are different from the actual rate of change of magnetic field as shown in Figure 13. Upon utilizing Equation (44) as measurement errors for the EKF, the gyroscope biases are successfully estimated and compensated from the gyroscope measurements bringing the estimated rate of change of magnetic field components in harmony with the actual ones, as shown in Figure 14.

**Figure 15.**
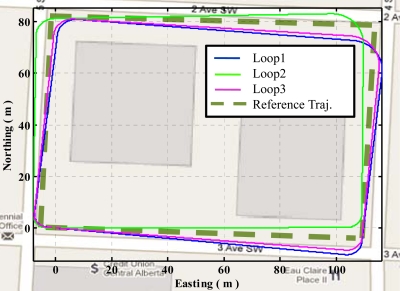
Trajectories obtained using QSF in urban canyon.

Figure 15 shows the three trajectories obtained using QSF measurements in the urban canyon. These trajectories are obtained by initializing the starting position and orientation for each loop. The first observation is the consistency of the ending locations. These are within 2 m of one another showing the effectiveness of QSF in estimating the rate gyroscope errors. The other observation is the random skewing of the three trajectories with respect to one another. This is because the QSF measurements can completely observe the rate gyroscope errors, but are not capable of observing the actual attitude errors. The attitude error growth is constrained using QSF measurements as is evident from Equation (49). As the rate gyroscope errors are randomly varying, these cause random errors in the attitude at the beginning of each path while the gyroscope errors are being estimated, which results in a random orientation error. Once the rate gyroscope errors are completely estimated, the attitude error growth is constrained. Thus the accuracy of the estimated trajectory is improved through the use of the QSF detections.

**Figure 16.**
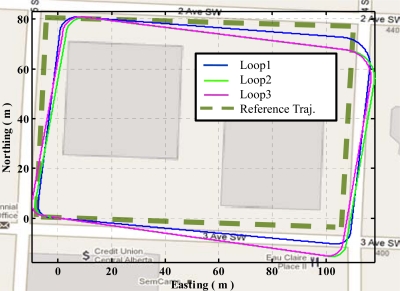
Continuous trajectory in urban canyon using QSF.

Figure 16 shows the trajectory obtained using the three loops in a continuous fashion. It is quite evident that Loop2 and Loop3 are very similar in this case. This is because the rate gyroscope errors have been properly estimated resulting in trajectories with a steady skew, which means that the orientation errors are now effectively constrained.

### 8.4. Performance of Attitude Estimator in Urban Environment

The maximum trajectory error obtained using raw magnetic heading based on measurement models for estimating continuous trajectory, whose length was longer than 1 km (for the three loops) is approximately 87 m whereas that for the QSF measurement model is approximately 16 m. Thus the trajectory errors are reduced by 80% by utilizing QSF measurements for constraining the attitude error growth and estimating the gyroscopes’ errors.

## 9. Conclusions and Future Work

This article investigated the use of handheld devices (smart-phones) equipped with low cost consumer grade sensors for pedestrian navigation. As the attitude/orientation errors play a major role in the overall navigation error budget, the focus was on improving the attitude estimates in environments where GPS is denied. For this purpose, the use of the Earth’s magnetic field as a measurement source for estimating the errors associated with low cost inertial sensors was investigated.

A novel method for utilizing the Quasi-Static magnetic Field (QSF), regardless of perturbation, to mitigate gyroscope errors has been developed and the corresponding equations have been presented. The novel estimation technique proved to deliver a high level of performance, reducing the trajectory errors by 80% for a distance of more than 1 km. This method detected the QSF periods during pedestrian’s motion and related the changes in the magnetic field components during these periods with the angular rates of the sensor block, thus providing measurements for directly assessing the errors associated with the rate gyroscopes. Development of an Extended Kalman Filter (EKF) for modeling the attitude and gyroscope errors as well as relating these to the magnetic field measurements have been conducted. They gave an insight into the interdependence of different parameters, which proved beneficial in identifying the limitations of the proposed model.

Selection of an urban canyon (magnetically disturbed outdoor field) for the experimental assessments provided data sets for a realistic and detailed analysis of the performance of the proposed algorithm. Analyzing the results in the position domain with the sensor platform carried in a hand gave detailed insight into the impact of the attitude estimator on the position error budget. A high accuracy wheel encoder made it possible to isolate the attitude errors from the position ones.

Even with an 80% error reduction in the positioning domain as compared with classical integration of magnetometers and gyroscopes in attitude estimation filter, the use of QSF measurements to successfully estimate the gyroscope errors resulted in constant orientation errors. This showed that this scheme is not sufficient for observing the absolute attitude errors. The position errors reached 16 m for a traversed trajectory of more than 1 km.

The use of accelerometers and pressure sensors for estimating pedestrian’s position as well as speed is necessary to completely assess the impact of this research in real world navigation scenarios. Both of these sensors are already incorporated in the MSP developed for this research and will form the basis for the above suggested research.

Research into the resolution of the ambiguity between sensor and body frames, which was constrained to be zero for the experiment herein, is needed. This can be achieved by using the accelerometers, but requires detailed modeling of the pedestrian’s walk related to arm swing or hip joint motion. The algorithm developed herein is self-contained and assumed a fully denied GNSS environment. However, GNSS is partly available in urban canyons and in numerous indoor environments. Hence research into the integration of the two approaches to maximize availability and accuracy is in order.

## Figures and Tables

**Table 1.** Parameters selected for the QSF detector.

**Parameter**	**Value**
Window Size (N)	3
Probability of detection (P_d_)	0.82
Probability of false alarm (P_f_)	0.30
Threshold (γ)	0.14
