# Correction: Renaudin, V. *et al.* Use of Earth's Magnetic Field for Mitigating Gyroscope Errors Regardless of Magnetic Perturbation. *Sensors* 2011, *11*, 11390-11414

**DOI:** 10.3390/s120608437

**Published:** 2012-06-20

**Authors:** Muhammad Haris Afzal, Valérie Renaudin, Gérard Lachapelle

**Affiliations:** Department of Geomatics Engineering, Schulich School of Engineering, University of Calgary, 2500 University Drive NW, Calgary, AB T2N 1N4, Canada; E-Mails: haris.afzal@ucalgary.ca (M.H.A.); gerard.lachapelle@ucalgary.ca (G.L.)

Although the equations' derivation in our paper published in Sensors 2011 [1] is correct, a typo has been found in the summarizing Equations (48) and (49). The dot on the B in the skew matrix should be removed. Equations (48) and (49) should be corrected as:

(48) $$\delta {\dot{\text{B}}}_{QSF_{k+1}}^{b}=\left[\begin{array}{cc} 0_{3\times 3} & -\left[{\text{B}}_{QSF_{k+1}}^{b}\times \right] \end{array}\right]\;\left[\begin{array}{c} {ɛ}^{n}\\ \delta {\text{b}}_{\omega } \end{array}\right]+\left[\begin{array}{cc} 0_{1\times 3} & 0_{1\times 3}\\ 0_{1\times 3} & \left[{\hat{\omega }}_{\text{B}}^{b}\times \right] \end{array}\right]\;\left[\begin{array}{c} 0_{3\times 1}\\ \eta_{\text{B}} \end{array}\right]$$

(49) $$\underset{\delta \text{z}}{\underset{︸}{\left[\begin{array}{c} \delta {\text{B}}_{QSF_{k+1}}^{n}\\ \delta {\dot{\text{B}}}_{QSF_{k+1}}^{b} \end{array}\right]}}=\underset{H_{\text{QSF}}}{\underset{︸}{\left[\begin{array}{cc} -\left[{\text{B}}_{QSF_{k}}^{n}\times \right] & 0_{3\times 3}\\ 0_{3\times 3} & -\left[{\text{B}}_{QSF_{k+1}}^{b}\times \right] \end{array}\right]\;}}\underset{\delta \text{x}}{\underset{︸}{\left[\begin{array}{c} {ɛ}^{n}\\ \delta {\text{b}}_{\omega } \end{array}\right]}}+\left[\begin{array}{c} -{\text{C}}_{b}^{n}\\ {\hat{\omega }}_{B}^{b} \end{array}\right]\eta_{B}$$
